# SWIEET—a salt-free alternative to QuEChERS

**DOI:** 10.1007/s00216-024-05525-0

**Published:** 2024-09-18

**Authors:** Nadja Kalinke, Pascal Stopper, Luca Völkl, Florian Diehl, Carolin Huhn

**Affiliations:** Department of Chemistry, Institute of Physical and Theoretical Chemistry, Eberhard Karls Universität Tübingen, Tübingen, Germany

**Keywords:** Electroextraction, Phase separation, Sugaring-out, Polar analytes, Miscibility gap

## Abstract

The efficient extraction of various analytes from a wide spectrum of matrices with organic solvents is still a great challenge in analytical chemistry. Especially polar and charged compounds are hard to extract in combination with neutral analytes of intermediate to low polarity. The QuEChERS method is often chosen and has been adapted not only to the analysis of food samples, but also to environmental matrices (soil, wastewater) or biota. In this study, we overcome major drawbacks of QuEChERS such as low recoveries of charged analytes and impairment of downstream analysis by high salt loads. The new extraction method, applicable to liquid and solid samples, is called SWIEET (sugar water isopropanol ethyl nitrile extraction technique). Phase separation of the otherwise miscible extraction solvents water and acetonitrile is achieved by sugaring-out instead of salting-out. Extraction efficiencies were greatly improved by adding isopropanol to the acetonitrile phase. The concentrations of the additives glucose and isopropanol, as well as temperature, were optimized by a design of experiment. Further improvement was achieved through electro- or double-extractions. For all sample types tested (surface water, wastewater treatment plant effluent, tomato, soil, and oats), recoveries and precision were higher with SWIEET than with the established QuEChERS method. From wastewater treatment plant effluent, 75% recovery on average were achieved with our SWIEET method compared to 37% with QuEChERS for a model analyte mixture with polarities of logD_pH7_ =  − 5.7 − 3.5. Higher recoveries and lower standard deviations compared to QuEChERS were achieved especially for polar and charged analytes such as metformin. Handling proved to be easy, since there was no additional solid phase and no tedious weighing of salts.

**Graphical Abstract:**

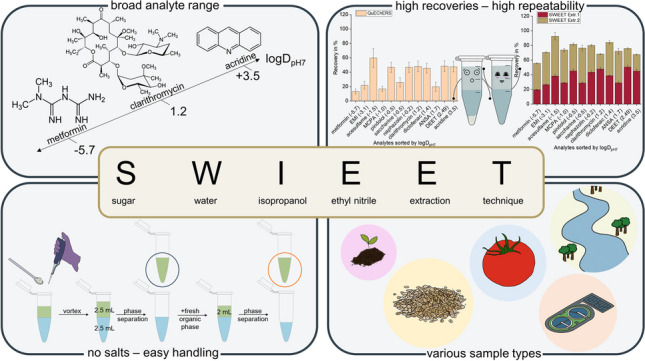

**Supplementary Information:**

The online version contains supplementary material available at 10.1007/s00216-024-05525-0.

## Introduction

The extraction method QuEChERS was first published in 2003 by Anastassiades et al. [1]. Since then, it has become the gold standard for the extraction of a broad spectrum of analytes from many different types of samples [2]. The method is applicable to solid and liquid samples and uses two liquid phases with their separation being induced by salts, often followed by a cleanup using dispersive solid-phase extraction (dSPE). Parameters like pH, solvent composition, and salt concentration were optimized for different target analytes and matrices, enabling a wide range of applications, as reviewed by Perestrelo et al. [3]. A major disadvantage of QuEChERS is the high amount of salt used during extraction, which can impair downstream analysis. Especially in ESI–MS, ion suppression can occur [4], as well as increased formation of sodium adducts. Furthermore, classical QuEChERS salts like NaCl and MgSO_4_ are not volatile and therefore form deposits on surfaces of the analytical instrumentation [5]. The p-QuEChERS method uses potassium phosphates instead of the classical QuEChERS salts, so problems related to solid salt phase remain, but the formation of magnesium complexes is avoided and recoveries can be increased [6]. The salts used for the extraction have to be weighed for each sample, which is time-consuming. The other option is to buy QuEChERS kits, which significantly increases the costs of the sample preparation and limits possibilities to adapt the method to the analytical task under consideration. A further development of QuEChERS is QuEChERSER, which was introduced to further broaden the polarity range using acetonitrile/water for extraction first. A few µL are used for direct LC–MS analysis, then a salting-out step is used to create an extract for GC–MS analysis [7–9]. An orthogonal method to QuEChERS is QuPPe. Here, acidified methanol is used for the extraction of very polar analytes, while non-polar analytes are hardly recovered [10].

Alternative strategies published so far replaced salts by sugars or organic solvents or lowered the temperature to reach the miscibility gap of water and acetonitrile.

### Salting-out

The concept of salting-out has been known for centuries and used in organic chemistry for sample workup but also in larger scale industrial purification processes or smaller scale purification of proteins [11]. The solvent system in QuEChERS consists of acetonitrile and water. As acetonitrile and water are fully miscible, a miscibility gap has to be introduced, for which QuEChERS uses salts, mainly NaCl and MgSO_4_. These salts are strongly bonded to water, so that a solvent layer forms around the salt ions [11]. This decreases the solubility first of all for acetonitrile in water until the miscibility gap is reached. The same process is relevant for many other organic compounds and can be used to enrich analytes in the organic acetonitrile phase for further analysis [12].

### Temperature-induced phase separation

For samples with a high fat content, lowered temperatures were used together with salt addition to evoke phase separation. At low temperature, lipids precipitate and can easily be removed [13]. As a QuEChERS alternative, salts may even be omitted and phase separation induced just by lowering temperatures as shown by Shao et al. [14] who named the method cold-induced aqueous acetonitrile phase separation (CIPS-QuEChERS). Similarly, to remove acetonitrile from otherwise aqueous protein solutions, Gu et al. [15] used low temperatures to induce a phase separation. The concept of adapting temperature to manipulate the miscibility gap was further demonstrated by Ullmann et al. [16], who extracted dyes in hexane-methanol and water-acetonitrile-toluene mixtures. The phase composition changed continuously by heating or cooling the mixtures. This is advantageous as no transfer across a sharp phase boundary is required upon the continuous formation of the two-phase system. A great advantage is that less reagents are needed for this method. However, robustness of phase composition and thus repeatability of the extraction are compromised if the temperature cannot be fully controlled.

### Sugaring-out

Wang et al. [17] described the induction of a phase separation by addition of sugar to an acetonitrile–water system. Considering the influence of temperature on the ternary system, phase diagrams were also recorded to optimize acetonitrile retrieval after HPLC [18, 19]. Sugars neither interact with analytes nor change the sample solution in terms of pH contrary to the salts used in QuEChERS [3], which can facilitate robust analyte extraction. The influence of different sugars and polysaccharides on the distribution coefficients of various acidic compounds was investigated [17]. Their distribution coefficients between the organic and aqueous phase ranged between 1.7 and 8.9. For the polar vanillin [20, 21], extraction efficiencies up to 95% were reported. Using sugar also enabled the use of electroextraction, which is not possible in samples with high salt loads due to the high conductivity. For example, Mahdavi et al. [22] observed an improvement of the extraction efficiency, a stabilization of the current and lowered electrolytic reactions using sugars for the electromembrane extraction of basic drugs through a supported liquid membrane. Sugars, just like salts, are cheap and readily available. Since sugars are neutral, sugaring-out can be expected to be compatible with most downstream analytical methods which are sensitive to salt loads such as capillary electrophoresis and HILIC. Finally, the main advantage of sugar additives is their high solubility, resulting in facilitated method development and reduced workload, because pipetting highly concentrated sugar solutions is possible instead of weighing salts for each sample.

### Organic solvents as additives

Another method for extractions based on phase separation was suggested by Gupta et al. [23] who induced phase separation adding an organic modifier to the mixture of acetonitrile and water. They used methyl isobutyl ketone as a modifier in addition to sodium chloride. Hydrophobic solvents were often used as modifiers: e.g., Liu et al. [24] used non-oxygenated solvents, such as dichloromethane (DCM), as modifiers in an acetonitrile–water system to extract flavonoids from plants, achieving higher recoveries than by using salts alone. They also applied their method to plasma samples spiked with three model drugs [25]. Hu et al. [26] tested ethyl acetate (EtOAc), ethyl ether, and methyl tert-butyl ether as modifiers for the extraction of 17 organophosphate flame retardants and plasticizers from urine, achieving higher recoveries in comparison to solid-phase extraction. The main downside is that many of the modifiers used are toxic. To our knowledge, polar protic solvents have not yet been used as modifiers in an acetonitrile–water system. Advantages of this method compared to classical salting-out include facilitated and faster execution and the ease to adapt phase ratios and phase composition.

### Electroextraction

Using electric fields, extraction of charged analytes can be enhanced from various liquid samples. This can be done across a solid or liquid membrane or directly across one or more phase boundaries [27–29] present in mixtures with miscibility gaps. However, this is only possible if salts are avoided to keep the conductivity low. Only a few of the strategies of inducing a phase separation described so far are compatible with electroextraction. As discussed previously, temperature plays a significant role for extraction, which may, however, be in conflict with temperature changes that occur during electroextraction due to Joule heating. Continuous cooling would be needed during the experiments to prevent disintegration of the phase boundary if the phase ratio and composition strongly depended on temperature. This would cause the need for a complex instrumental setup to ensure isothermal conditions. Furthermore, electrophoretic mobilities decrease at low temperatures.

With regard to the choice of organic solvents, their permittivity needs to be considered: If the permittivity is too low, no stable electric field can form and ion pair formation may prevent electromigration. In literature, next to 1-pentanol [30–32], EtOAc [33–37] is the most frequently used organic solvent for electroextraction, either as free liquid membrane or donor phase. It fulfils the requirement of immiscibility with water, but its permittivity is relatively low, so acids or other electrolytes are added to increase conductivity [37]. Sugaring-out systems are a suitable option for electroextraction as sugars do not impair the electric field, but they may reduce electrophoretic mobilities by increasing the viscosity of solutions.

In this study, we developed a new extraction method for a wide range of analytes, which is applicable to a broad spectrum of sample types. Overcoming the downsides of salt used to induce a miscibility gap, we chose a sugaring-out approach. A focus of this work was to improve the recoveries of polar analytes by addition of polar solvents to the extraction mixture. To further improve the extraction of charged analytes, electroextraction and double-extractions were envisaged. QuEChERS extractions were made for comparison.

## Materials and methods

### Chemicals

1-Ethyl-3-methyl-imidazolium (EMI, ≥ 95%), 2-methyl-4-chlorophenoxyacetic acid (MCPA), 5-amino-2-naphthalene sulfonic acid (ANSA, ≥ 95%), acesulfame (ACE, ≥ 99%), acridine (ACR, 97%), alpha-D-glucose (96%), carbon (> 99%), clarithromycin (CLA, ≥ 98%), di-(2-ethylhexyl)phosphoric acid (DEHPA, ≥ 98%), dextran 450,000–650,000, diclofenac sodium salt (DIC, ≥ 98%), isopropanol (iPr, LC–MS grade), magnesium sulfate, methanol (MeOH, LC–MS grade), naphazoline (NAPHA, ≥ 98%), pindolol (PIN, 98%), polyethylene glycol 6,000, poly(4-styrensulfonic acid), poly(vinyl alcohol) 31,000–50,000, poly(vinyl alcohol) 89,000–98,000, poly(vinyl alcohol) 146,000–186,000, potassium phosphate monobasic (≥ 99.5%), saccharine (SAC, ≥ 98%), and tert-butyl alcohol (≥ 99%) were purchased from Sigma-Aldrich (Steinheim, Germany). 4-Hydroxybenzoic acid (HBA, ≥ 98%), chloroform (> 99%), L-proline (≥ 99%), *N*-diethyl-m-toluamide (DEET, ≥ 98%), polyethylene glycol 35,000, poly(vinyl alcohol) 22,000, poly(vinyl alcohol) 72,000, poly(vinyl alcohol) 100,000, and sodium chloride (p.a.) were from Fluka (Buchs, Switzerland). Aliquat 336 TG (≥ 90%), dichloromethane (DCM, LC–MS grade), D( +)-galactose (≥ 99%), ethyl acetate (EtOAc, p.a.), and water (LC–MS grade) were provided by Thermo Fisher (Kandel, Germany). Acetonitrile (MeCN, LC–MS grade), D( +)-xylose (≥ 99%), formic acid (> 99%), glycerol (≥ 98%), indigo carmine, and tri-sodium citrate dihydrate (≥ 99%) were bought from Roth (Karlsruhe, Germany). Metformin (MET, 97%) was from Alfa Aesar (Haverhill, MA, USA), trehalose dihydrate (97%) from BLDpharm (Shanghai, China), dextran sodium sulfate 40,000 from ICN Biomedicals (Aurora, OH, USA), D-sorbitol (97%) from Acros Organics (NJ, USA), C18 and PSA from Agilent (Waldbronn, Germany), and polyethylene glycol 600 and 1000 from Merck (Darmstadt, Germany). Doubly-distilled water was produced using a PURELAB Classic PL5241 (ELGA LabWater, Celle, Germany).

#### Model analyte mix

To cover a broad spectrum of analytes regarding size, polarity, charge, and functional groups, 13 model analytes were chosen to optimize and judge the performance of the extraction protocol (Table 1). Equal amounts of the analytes’ methanolic stock solutions (1 g/L) were mixed to obtain the analyte mix at an individual analyte concentration of 77 mg/L each. For extraction experiments, the concentration of analytes in the aqueous mixture was 3 mg/L.

**Table 1:** Model analytes, their charge number at pH 7 and logD_pH7_

Analyte	Charge number_pH7_	logD_pH7_
Metformin	+ 2	− 5.7
1-Ethyl-3-methyl-imidazolium	+ 1	− 3.1
Pindolol	+ 1	− 0.5
Naphazoline	+ 1	− 0.2
Clarithromycin	+ 1	1.2
Acridine	0	3.5
5-Amino-2-naphthalene sulfonic acid	0	1.7
*N*-Diethyl-*m*-toluamide (DEET)	0	2.5
4-Hydroxybenzoic acid	− 1	− 1.2
Diclofenac	− 1	1.4
Acesulfame	− 1	− 1.5
MCPA	− 1	− 1
Saccharine	− 1	− 0.5

### Extraction procedure

#### Spiking of dry samples

For solid and dry samples, such as soil and oats, 0.98 mL of the analyte mix were added per 20 g sample, as well as 20 mL doubly distilled water to ensure proper distribution of the analytes on the sample. The mixture was shaken for 1 h using an overhead shaker. The water was evaporated from the sample at 60 °C, so analytes could sorb on the sample surface.

#### QuEChERS extraction

Aqueous samples: Analyte Mix was spiked to 2.5 mL aqueous sample such as surface water or wastewater treatment plant effluent to a final concentration of 3 mg/L. After adding 2.5 mL MeCN to the aqueous sample, the extraction mixture was mixed for 1 min using a vortexer. To the mixture, 0.25 mg NaCl and 1 mg MgSO_4_ were added, and the slurry was immediately shaken vigorously for another minute. The extraction mixture was centrifuged at 3500 rpm for 10 min. Phases were separated by pipetting the upper phase into a different vial.

Dry samples: To 1.5 g of the spiked and dried sample, 2.5 mL doubly distilled water and 2.5 mL MeCN were added, and the extraction mixture was mixed for 1 min using a vortexer. To the mixture, 0.25 mg NaCl and 1 mg MgSO_4_ were added and immediately shaken vigorously for another minute. The extraction mixture was centrifuged at 3500 rpm (1233 rcf) for 10 min. Phases were separated by pipetting the upper phase into a different vial.

For double-extractions, another 2 mL fresh MeCN was added to the residual aqueous phase, and the extraction mixture was mixed for 1 min using a vortexer and centrifuged at 3500 rpm (1233 rcf) for 10 min. Phases were separated again by pipetting the upper phase into a different vial.

An aliquot of each or the combined organic phases diluted for LC–MS analysis (see “Quantification”) and directly analyzed or stored at -20 °C.

#### Design of experiment

For the design of experiment (DoE) the software Develve Version 4.14.0.0 (Velp, the Netherlands) was used for design, calculation, and plotting of the Box-Behnken design.

#### Optimization of the SWIEET extraction protocol

Various additives were added at different concentrations to an aqueous sample, containing 3 mg/L analyte mix: 2.5 mL of this spiked sample was mixed with the same volume of organic extraction mixture consisting of acetonitrile and 5–20 vol.% of either EtOAc, isopropanol, DCM, or chloroform. The mixture was homogenized for 1 min using a vortexer. After a clear phase boundary was visible, usually within a minute, phases were separated by pipetting.

For double-extraction, 1–2.5 mL fresh organic extraction mixture consisting of acetonitrile with either 10 or 20 vol.% isopropanol was added to the aqueous phase. After mixing for 1 min using a vortexer, the phases were allowed to separate again.

An aliquot of the organic phase was diluted with methanol for LC–MS analysis (see “Quantification”) and then stored at − 20 °C or directly analyzed.

#### Final SWIEET protocol

Aqueous samples: For aqueous samples, glucose was added to the sample to a final concentration of 2 M, containing 3 mg/L analyte mix. A mixture of 2.5 mL of this spiked sample and the same volume of organic extraction mixture consisting of 80 vol.% acetonitrile and 20 vol.% isopropanol was prepared. The mixture was homogenized for 1 min using a vortexter. After a clear phase boundary was visible, usually within a minute, phases were separated by pipetting.

Dry samples: To 1.5 g of the spiked and dried sample, 2.5 mL of a 2 M aqueous glucose solution and 2.5 mL organic extraction mixture, consisting of 80 vol.% acetonitrile and 20 vol.% isopropanol, were added. The mixture was homogenized for 1 min using a vortexer. After a clear phase boundary was visible, phases were separated by pipetting.

For double-extraction, 2.5 mL fresh organic extraction mixture consisting of 80 vol.% acetonitrile and 20 vol.% isopropanol were added to the residual aqueous phase from the first extraction step. After mixing for 1 min using a vortexer, the phases were allowed to separate again. Organic phases of the two extraction steps were combined prior to analysis.

An aliquot of the organic phase was diluted with methanol for LC–MS analysis (see “LC–MS sample preparation”) and then stored at − 20 °C or directly analyzed.

#### LC–MS sample preparation

An aliquot of 10 µL of the organic extract was diluted with 40 µL MeOH for RPLC-MS analysis. Matrix-matched calibration was used to quantify the analytes. For this, the organic phase from a blank extraction (using doubly distilled water as a sample) was spiked at four concentration levels with all analytes.

#### Electroextraction

For electroextraction, a standard 5 mL plastic syringe was equipped with 0.5-mm-thick and 5-mm-long platinum electrodes at the outlet and the stamp, connected to a voltage source (Keithley 2290E5, Keithley, Cologne, Germany). The two-phase system was transferred to the syringe after mixing for 1 min using a vortexer. A constant current of 200 µA (chosen after optimization) was applied for 10 min. The aqueous and organic phases were collected in separate tubes.

### LC–MS method

The RPLC-MS method was adapted from Rösch et al. [38]. A 1260 Infinity LC system coupled to a 6550 iFunnel Q-TOF (Agilent Technologies, Waldbronn, Germany, or Santa Clara, CA, USA) was used. An aliquot of 2 µL of the diluted sample was injected onto a Zorbax Eclipse Plus C18 column (2.1 × 150 mm, 3.5 µm, Agilent Technologies) equipped with a Zorbax Eclipse Plus C18 guard column (2.1 × 12.5 mm, 5 µm, Agilent Technologies). The mobile phase consisted of water and acetonitrile with 0.1% formic acid. A gradient was used at a flow rate of 0.3 mL/min. Initially, a water content of 95% was used for 1 min, and then it was decreased to 5% over 7 min and held for another 7 min. Finally, the water content was increased to 95% for 5 min.

For MS analysis, a jet-stream electrospray ionization source was used. The nebulizer pressure was 35 psig, drying gas temperature 160 °C, drying gas flow rate 16 L/min, fragmentor voltage 360 V, capillary voltage + / − 4000 V, skimmer voltage 65 V, and nozzle voltage 500 V. The sheath gas had a temperature of 325 °C and was used at a flow rate of 11 L/min. Spectra were acquired at a rate of 1 spectrum/s in the mass range of 40–1000 m/z. Solutions of purine and HP0921 (Agilent Technologies) in methanol/water (95/5) were constantly infused into the ESI source through a reference sprayer for internal calibration.

Extracted ion chromatograms of the model analytes acquired with this method are shown in Fig. S2 in the supporting information.

### Quantification

The phase ratio was determined weighing the separated phases (m(org) and m(aq)). Analyte concentrations from the samples were calculated using the calibration curve resulting from matrix-matched calibration, taking dilution into account. Recoveries were calculated using the concentrations determined in the organic phase after extraction (c(org)) by LC–MS, the starting concentration in the aqueous sample (c(aq)), and the phase ratio after the extraction (m(org)/m(aq)):

1 $$rec\%=\frac{c(org)}{c(aq)}\bullet \frac{m\left(org\right)}{m\left(aq\right)}\bullet 100$$

Average recoveries of extractions (*n* = 3 or *n* = 5) were determined for individual analytes. For an easier comparison of different extraction protocols, medians and averages over all 13 model analytes were calculated. In addition, analytes were grouped into polar (logD_pH7_ < 0) and unpolar (logD_pH7_ > 0) substances.

For the determination of matrix effects (ME), the average peak area of five post-extraction spiked blank extracts (A) was compared to the average peak area of five spiked reference samples (B) of the analytes in methanol using Eq. (2):

2 $$ME\%=100-\frac{A}{B}\bullet 100$$

Positive values indicate ion suppression, and negative values indicate ion enhancement.

## Results and discussion

### Temperature-induced phase separation

Color experiments were used in this study to monitor and visualize the robustness of the phase separation. Since the phase composition has a great influence on the extraction recovery, temperature has to be taken in account when optimizing extraction parameters. All liquid–liquid extractions across a phase boundary are based on miscibility gaps of a mixture of at least two solvents. The width of this miscibility gap defining the composition of the two (mixed) phases can vary with temperature. For example, in a water-acetonitrile-EtOAc mixture, Takahashi et al. [39] showed that the miscibility gap is broader at 0 °C than at 25 °C. This means that at a fixed composition of the mixture, the organic phase contains less water and the aqueous phase less organic solvent at 0 °C. Thus, the polarity difference between the two phases is enhanced. To visualize this, we added indigo carmine as a model analyte to a two-phase system from water, acetonitrile, and EtOAc. The dye is well soluble in water, but hardly in the (pure) organic solvents. It is thus mainly present in the aqueous phase but in the organic phase only when its water content is high. Indigo carmine can thus be used as a marker of the water content in the organic phase.

**Fig. 1:**
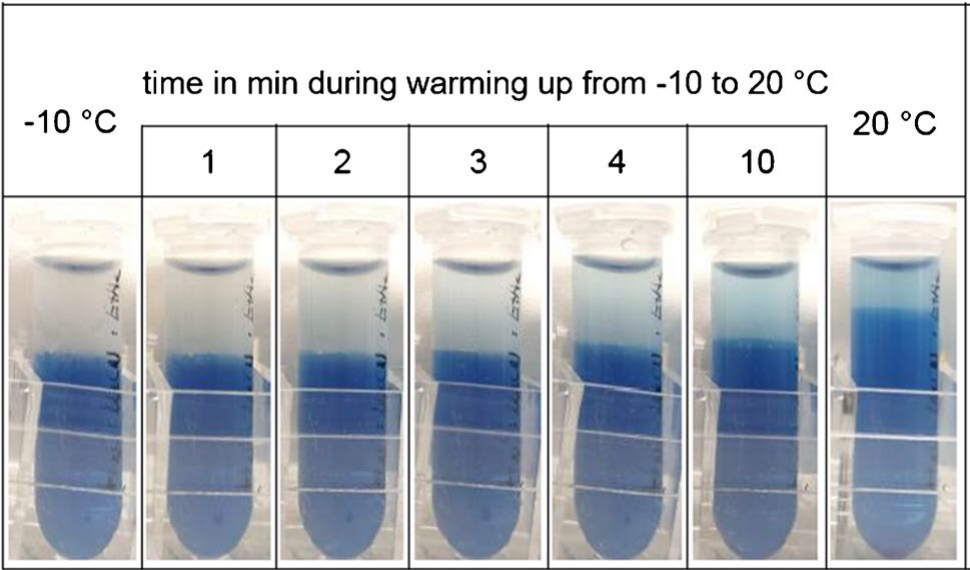
Photos of a two-phase system from a water-acetonitrile-EtOAc (50–40-10) mixture with 0.1 g/L indigo carmine at different time points while warming from − 12 °C (*t* = 0 min) to 20 °C (*t* = 20 min)

Figure 1 shows that at − 12 °C, a stable two-phase system is present with EtOAc added as an organic modifier. The upper phase is almost colorless, indicating rather pure phases with a low water content in the organic phase. Upon warming the mixture, the phase boundary becomes more diffuse, and the mole fraction of indigo carmine in the organic phase increases, turning it deeper blue, whereas the aqueous phase lightens. This observation shows that at 20 °C, the phase composition chosen for the experiment must lie close to the upper critical point of the miscibility gap. Due to the enhanced mixing at higher temperatures, the water content in the organic phase, and therefore polarity, increases. The two phases become more similar in their characteristics. Additionally, the phase volume changes upon warming. The volume of the aqueous phase increases, indicating a higher content of organic solvent compared to the mixture at − 10 °C. Since polarity and volume of the organic phase have a great influence on which analytes are preferentially extracted, extractions can be controlled by the water content in the organic phase and thus also by temperature.

### Screening of additives to induce phase separation

To avoid the problems salts can cause in downstream analysis, we investigated a salt-free approach to induce a stable phase separation. Most two-phase systems rely on water and acetonitrile, since it is compatible with gas and liquid chromatography, as well as SPE, and because toxicity and environmental relevance are relatively low. Other frequently used solvents for extraction are acetone and EtOAc, but phase separation is easier with acetonitrile than with acetone, and polar analytes were better extracted than with EtOAc [1]. To support phase separation, 20% EtOAc was added to the acetonitrile–water system, though it does not induce a stable and complete phase separation at ambient temperature, as seen in Fig. 1. In a screening approach, possible additives were chosen due to their previous use in liquid–liquid extractions or their number of OH-groups, which can bind water and therefore improve phase separation [22]. Molarities were chosen following Cray et al. [40] or based on own pre-studies and limiting factors like solubility. The additives were judged by their ability to induce a stable phase boundary and, when successful, by the median recovery for all model analytes (Table 2).

**Table 2:** Additives screened for phase separation at the molarity stated and the temperature, at which phase separation was observed. Median recoveries were determined for 13 model analytes (see the “Model analyte mix” section) by LC–MS analysis of the organic phase after extraction. Extractions were conducted by adding 2.5 mL organic extraction mixture consisting of 20% EtOAc and 80% acetonitrile to 2.5 mL of doubly distilled water spiked with 3 mg/L analyte mix

Additive	Molarity in mmol/L	Phase separation temperature	Median recovery in %
Aliquat 336TG	89.07	− 10 °C	x
Dextran 450 k–650 k	0.00727	a.t	33
Dextran 450 k–650 k	0.0726	a.t	x
Dextran sodium sulfate 40 k	0.994	a.t	35
Di-(2-ethylhexyl)phosphoric acid	100.6	a.t	46
D-Sorbitol	608.1	a.t	28
Galactose*	1000	a.t	27
Glucose*	1000	a.t	28
Glycerol	92.1	a.t	4
L-Proline	719.2	a.t	24
Polyethylene glycol 600	56.2	− 10 °C	41
Polyethylene glycol 1000	3.304	− 10 °C	x
Polyethylene glycol 1000	148.0	− 10 °C	x
Polyethylene glycol 6000	0.631	− 10 °C	x
Polyethylene glycol 6000	24.67	− 10 °C	x
Polyethylene glycol 35 k	0.114	− 10 °C	x
Poly(4-styrene sulfonic acid)	0.245	− 10 °C	x
Poly(vinyl alcohol) 22 k	0.183	− 10 °C	x
Poly(vinyl alcohol) 31 k–50 k	0.0985	− 10 °C	x
Poly(vinyl alcohol) 72 k	0.0554	− 10 °C	x
Poly(vinyl alcohol) 89 k–98 k	0.0427	− 10 °C	x
Poly(vinyl alcohol) 100 k	0.0400	− 10 °C	x
Poly(vinyl alcohol) 146 k–186 k	0.0241	− 10 °C	x
Potassium phosphate (monobasic)	102.6	a.t	25
tert-Butyl alcohol	97.14	− 10 °C	x
tert-Butyl alcohol	974.1	− 10 °C	33
Trehalose*	1000	a.t	41
tri-Sodium citrate dihydrate	99.78	a.t	29
Urea	1000	a.t	12
Xylose*	1000	a.t	22

a.t., ambient temperature; x, not selected for detailed investigation

^*^An organic extraction mixture with 35% EtOAc and 65% acetonitrile was used to ensure stable phase separation without additive for better comparison

With some of the additives, phase separation only occurred at lower temperatures. Since cooling during the extraction requires a more complex instrumental setup, these additives were not preferred for further optimization. Some of the additives that exhibited a phase separation at ambient temperature were chosen for further experiments. We included the liquids PEG600 and tert-butyl alcohol, as they promised easy handling, despite phase separation only occurring at − 10 °C. In the second step, the additives were added to the extraction mixture and recoveries of the model analytes in the organic phase were determined. Lowest median recoveries were achieved using glycerol and urea. Contrary to the kosmotropic salts that are commonly used in QuEChERS, glycerol and urea are chaotropic. These chaotropes aided phase separation, but did not enhance extraction efficiencies. The addition of dextran 450 k–650 k resulted in relatively high median recoveries, but due to its low solubility, precipitation occurred already at lower molarities during extraction, resulting in a lower repeatability. High recoveries were also achieved using PEG600, but in LC–MS analysis, large amounts of the polymer were detected in the extract with a partial signal overlap with analyte signals.

The class of additives that consistently yielded high recoveries were sugars. Details are presented in Section S2 in the supporting information. All of them are highly soluble in water, are non-toxic, and had no impact on LC–MS analysis. Therefore, glucose, galactose, trehalose, and xylose were further investigated as additives to improve phase separation and analyte recovery. At the higher EtOAc contents of 35% used for these experiments, phase separation was possible without sugar addition, enabling a direct comparison of the effects of the different sugars. As shown in Fig. S3, all sugars improved the average recovery compared to an extraction using only EtOAc as a phase separating additive. Best overall recoveries were achieved using trehalose (40%), glucose (33%), and galactose (32%), and the use of xylose resulted in slightly lower recoveries (29%). The recoveries for glucose and galactose, which have the same number of OH-groups, were very similar. Xylose has one OH-group less, and so the lowest recoveries determined here corroborate findings by Mahdavi et al. [22], who hypothesized that the number of OH-groups in a molecule is essential for the effectivity of sugaring-out. If more water is required for carbohydrate solubilization, the solubility of the analytes in the aqueous phase decreases, which increases recoveries. The underlying mechanism is an excluded volume effect [41], meaning that H-bonds in water are bound by carbohydrates and cannot take part in the in solubilization of the analytes. The analyte concentration then increases in the organic phase. As can be expected from this mechanism, the improvement of recoveries by using sugars is more pronounced for polar analytes, which is favorable knowing that polar analytes are usually harder to extract from aqueous samples. The QuEChERS method, for example, is mainly used for analytes with low to medium polarity (− 1 < logD_pH7.4_ < 7) [42]. Solubility of the analytes is not only based on the excluded volume effect, but also on the permittivity of the phases, which changes when a cosolvent is added [43]. For aqueous glucose solutions, the permittivity decreases with increasing glucose concentration [44]. Thus, a decrease in solubility would be expected for polar solutes in the aqueous phase. The combination of fewer available H-bonds and reduced permittivity could explain the increased polar analyte concentration in the organic phase.

However, recovery not only depends on concentration, but also the volume ratio of the two phases. The addition of sugars to acetonitrile-EtOAc mixtures increases the volume of the organic phase, which shifts the phase ratio and therefore recovery (see Eq. (1)). Importantly, glucose largely remained in the aqueous phase [19] (see also Section S2 in the supporting information) minimizing interferences in LC–MS analysis.

Unfortunately, recoveries were very low for the most polar analytes metformin and EMI (1% and 3%) and under 40% on average for all polar analytes, which shows that further optimization was necessary. For this, we chose to use sugars. Among them, glucose was preferred for its low price, good recovery, and high solubility.

### Choice of organic solvent

We tested several organic solvents chosen from literature studies, preliminary work, or theoretical considerations. We reached a stable phase separation when adding only sugars to water and acetonitrile. This allowed us to improve recoveries using solvents as additives, which are not able to induce phase separation when added alone. In first experiments, EtOAc was used as an additive in the organic phase. However, in preliminary studies, we observed that especially polar (logD_pH7_ < 3) and charged compounds were hardly extracted using this solvent. To improve recoveries for these analytes, an elevated polarity and permittivity of the organic phase, as well as the ability to form H-bonds, were envisaged adding the polar and protic isopropanol.

Recoveries improved through the addition of isopropanol instead of EtOAc for most analytes, but especially for the most polar analytes metformin and EMI. For these analytes, the addition of isopropanol also yielded higher recoveries compared to the halogenated solvents chloroform and dichloromethane tested. Details on the choice of organic solvents are presented in Section S1. Due to the higher recoveries, and the low toxicity resulting from the addition of isopropanol, we chose this polar protic solvent for further optimization of the extraction method.

### Optimization of extraction parameters

Further optimization was made with the addition of glucose and isopropanol to the water-acetonitrile mixture considering different temperatures. Conducting a design of experiment (DoE) allows to simultaneously vary multiple parameters in a manageable number of experiments. A Box-Behnken design was chosen because it avoids combinations of extreme values that would lead to instable phase separations. The center point of the DoE was conducted three times to ensure statistical significance. The ranges of the parameters were 0–25 °C, 5–10% isopropanol in 2.5 mL organic extraction mixture (90–95% acetonitrile), and 1.5–2.5 M glucose in 2.5 mL aqueous extraction mixture, consisting of 3 mg/L analyte mix in doubly distilled water. The results are shown in hypersurface plots in Fig. S4.

Isopropanol concentration had the greatest effect on analyte recovery, whereas recoveries were least sensitive to temperature changes, indicating high robustness for possible differences in ambient temperature. In general, high isopropanol content, low glucose concentration, and high temperatures revealed highest recoveries. The maxima of the hypersurfaces lie at the corners of the tested ranges, which indicate that an actual maximum would be found at more extreme parameters, where, however, phase separation will become instable or even impossible. For example, a high isopropanol content yielded high recoveries, but also compromised phase separation. This is due to isopropanol being a polar protic solvent like water, at high isopropanol content the two phases become too similar evoking miscibility. Even though the maximum of the surface plot was observed at a glucose concentration of 1.5 M, 2 M was chosen for further experiments, since this concentration provided a higher stability of the system and resulted in higher repeatability of recoveries, especially at high isopropanol contents of 20%. Decreasing the temperature facilitated phase separation and guaranteed a stable phase boundary. However, when adding 2 M glucose, phase separation was already very robust, and cooling of the extraction system was not necessary. In addition, the surface plots show that lower temperatures were disadvantageous for recoveries. This might be due to the higher viscosity of the solvents and slower diffusion of the target analytes or differences in the width of the miscibility gap. A precise temperature control below room temperature would increase time and costs of the extraction, so further work was conducted at ambient temperature.

To achieve high recoveries while maintaining high repeatability, 20% isopropanol and 2 M glucose were chosen for extraction. The extraction process was clearly improved, but recoveries of < 50% on average were still not convincing. Thus, two strategies were followed for further optimization: electroextraction and double-extraction.

### Electroextraction

Electroextraction can improve recoveries for charged analytes. Therefore, Pt electrodes were inserted at the stamp and outlet of a plastic syringe. The extraction mixture was drawn up into the syringe after mixing. After phase separation was visible, voltage was applied. The experiment was conducted in duplicates, using reversed polarities for 10 min each. Setting a constant current compared to a constant voltage proved to be more stable. Constant currents in the range of 100–2000 µA were tested, and 200 µA was optimal regarding average analyte recoveries (data not shown). If the current was lower, the effect of the electric field was too small, but if the current was higher, side effects became prevalent: Electrolysis led to strong pH changes in the aqueous phase since it was not buffered, which can cause analytes to become neutral. For example, a pH of 3.6 instead of 7 was measured in the aqueous phase after the extraction with 2000 µA. Acesulfame and MCPA are negatively charged (z =  − 1) at pH 7, but at pH 3.6, the charge number decreases to *z* =  − 0.28 or *z* =  − 0.81, respectively. This led to lower recoveries compared to extractions with 200 µA.

Due to the addition of sugar, phase separation was stable despite elevated temperatures occurring due to Joule heating at high currents. A comparison of an extraction conducted with and without the application of an electric field at 200 µA showed an average increase of the recovery by 18% (see Fig. S6a). The increase was more pronounced for analytes with high charge numbers like metformin, EMI, and saccharine. Interestingly, however, an improvement of the recovery was observed for all analytes tested, charged analytes, and neutral ones. An improved analyte migration cannot directly be caused by the electric field, but may be due to changes of the local environment of analytes. As previously explained, due to electrolysis of water, pH changes can occur. Some analytes may have gained charge, which were neutral at the starting pH of 7. Furthermore, if more analytes are transported through the phase boundary, viscosity of the aqueous solution might decrease and facilitate diffusion of neutral analytes. Due to Joule heating, temperature increases and thus viscosity and interfacial tension decrease [45]. This likely facilitated phase transfer.

The same protocol was applied to a wastewater treatment plant effluent sample (see Fig. S6b). Due to the higher conductivity of the sample, the resulting voltage was about ten times lower than in the doubly distilled water sample, resulting in a weaker electric field. As a result, recoveries were lower, which could not be overcome by increasing the current to up to 2000 µA.

All in all, electroextraction proved beneficial to improve recoveries especially for charged analytes and recoveries increased to over 60% for many analytes. However, for real samples with higher ionic matrix loads, recoveries were still insufficient with under 60%, as exemplified for wastewater treatment plant effluent in Fig. S6b in the supporting information.

### Double-extraction with/without electroextraction

Another or additional strategy to improve recoveries is double-extraction. For this, fresh organic phase was added to the aqueous phase remaining after the first extraction, both for SWIEET and QuEChERS for comparison. To directly consider matrix effects, we conducted these experiments with wastewater treatment plant effluent. Since hydroxybenzoic acid consistently yielded low recoveries (< 30%) for SWIEET and QuEChERS extraction, we suspected a problem in downstream analysis. Therefore, we did not include this analyte into the average recovery calculations for the following experiments in real matrices.

**Fig. 2:**
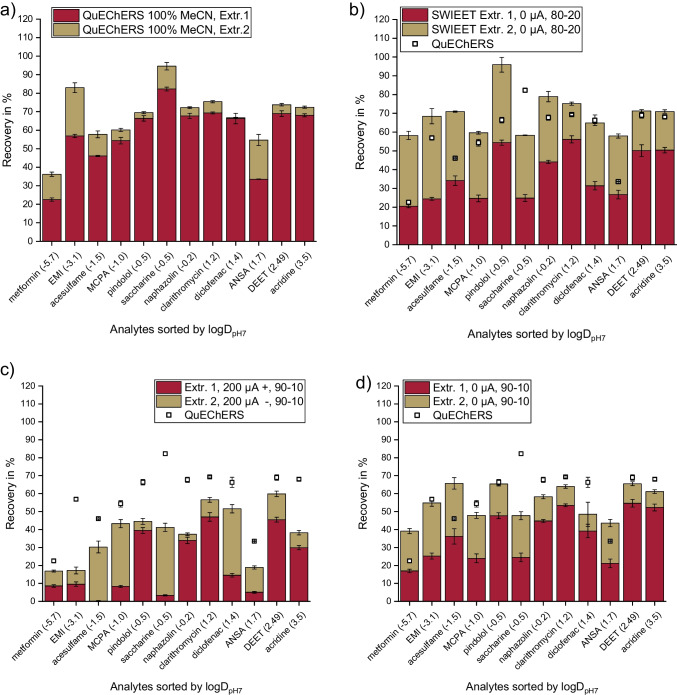
Excerpt of results for double-extractions according to Table 3 for ***a*** Exp. QuEChERS, ***b*** Exp. 9, ***c*** Exp. 4, and ***d*** Exp. 15. Recoveries of double-extractions of the analyte mix (see the “Model analyte mix” section) from 2.5 mL wastewater treatment plant effluent with ***a*** QuEChERS extraction (see the “QuEChERS extraction” section) and ***b***, ***c*** and ***d*** SWIEET extraction with 2 M glucose, ***c*** with 200 µA positive polarity in the aqueous phase in the first step and 200 µA negative polarity in the second step, and ***b*** and ***d*** without the application of an electric field in both steps. Composition of the organic extraction mixture for SWIEET: ***b*** 80 vol.% acetonitrile, 20 vol.% isopropanol (80–20); ***c***, ***d*** 90 vol.% acetonitrile, 10 vol.% isopropanol (90–10). Recoveries from QuEChERS Extr.1 are plotted also in ***b***, ***c***, and ***d*** for comparison. For detailed extraction protocols, see the “Optimization of the SWIEET extraction protocol,” “QuEChERS extraction,” and “Electroextraction” sections and Table 3

For QuEChERS double-extractions (Fig. 2a), recoveries were already relatively high in the first step with 57% on average. In the second step, however, only further 9% were extracted additionally (total average recovery = 66%). The increase was largest for polar analytes, e.g., for EMI, the recovery increased by 26%. Salt contents in QuEChERS are commonly above saturation; therefore, enough salt was left in the second step to induce a new phase separation, still with solid salt present as a third phase. No significant change in volume of the organic phase was observed between the two extraction steps. We thus assume that the compositions of the phases were similar in the second and first extraction step, explaining the low additional recoveries in the second step.

From the DoE (see the “Optimization of extraction parameters” section), we derived the optimized conditions for the SWIEET extraction, namely 2 M glucose and 20% isopropanol at room temperature. However, we decreased the isopropanol content to 10%, since this led to a more stable system. We observed that the volumes of the newly formed two phases differed significantly from the first extraction step: the volume of the organic phase increased significantly, which clearly indicates differences in the composition of the phases in the two extraction steps.

To understand the effects and to further increase recoveries from 47% on average, we varied the organic phase volume, organic phase composition, and the application of an electric field and its polarity for the first and second extraction in different combinations, resulting in 15 different protocols for double-extractions (see Fig. S7). Conditions and recoveries are summarized in Table 3.

**Table 3:** Average recoveries of 12 analytes obtained for the double-extractions (see the “Model analyte mix” section). Wastewater treatment plant effluent (2.5 mL) was spiked, and glucose was added at a final concentration of 2 M. The volume and the composition of the organic extraction mixture were varied. For electroextraction, different currents and polarities were used (“ + ” indicates positive polarity and “* − *” negative polarity applied at the electrode in the aqueous phase). QuEChERS extraction was conducted for comparison (Exp. Q). For the detailed extraction protocols, see the “QuEChERS extraction,” “Optimization of the SWIEET extraction protocol,” and “Electroextraction” sections

Nr	Step 1				Step 2				Total recovery
	Volume organic extraction mixture in mL	Composition organic extraction mixture (vol.% MeCN-vol.% iPr)	Current in µA and polarity	Average recovery in %	Volume organic extraction mixture in mL	Composition organic extraction mixture (vol.% MeCN-vol.% iPr)	Current in µA and polarity	Average recovery in %	Sum of average recoveries in step 1 + 2 in %
Q	2.5	100–0	x	57	2	100–0	x	9	66
1	2.5	90–10	200 −	19	2	90–10	200 −	16	34
2	2.5	90–10	200 −	21	2	90–10	200 +	19	40
3	2.5	90–10	200 −	21	2	90–10	x	30	52
4	2.5	90–10	200 +	20	2	90–10	200 −	18	38
5	2.5	90–10	200 +	23	2	90–10	200 +	10	33
6	2.5	90–10	200 +	20	2	90–10	x	28	48
7	2.5	90–10	x	35	2	90–10	200 −	13	48
8	2.5	90–10	x	32	2	90–10	200 +	10	43
9	2.5	80–20	x	37	2	80–20	x	32	69
10	2.5	80–20	x	31	2	90–10	x	31	62
11	2.5	90–10	x	33	2	80–20	x	26	60
12	2.5	90–10	x	35	2.5	80–20	x	9	44
13	2	90–10	x	13	2	90–10	x	32	45
14	2.5	90–10	x	22	1	90–10	x	14	36
15	2.5	90–10	x	37	2	90–10	x	18	55

Using an organic extraction mixture made of 90 vol.% acetonitrile and 10 vol.% isopropanol, we varied the relative volumes of the aqueous and organic phases. Adding the same amount of organic extraction mixture in both steps, 2.5 or 2 mL (experiments 12 and 13) resulted in lower recoveries of 44% and 45% in total for the two steps. This was lower compared to adding 2.5 mL in the first step and 2 mL in the second step, which yielded a total recovery of 55% (experiment 15). A reduction of the organic extraction mixture volume to 1 mL only in the second step was hypothesized to help enriching the analytes, but only 36% were recovered in total (experiment 14). It stands out that the robustness of the first extraction step is high, since the recoveries from the first step in experiments 7,8,11,12, and 15 varied only slightly at the same conditions (RSD = 5.7%). Only experiment 14 shows significantly lower recoveries and may be an outlier.

Varying the composition of the organic extraction mixture, highest average recoveries of 69% were achieved using an organic extraction mixture with 20% isopropanol and 80% acetonitrile for both extraction steps (experiment 9, Fig. 2b). After the addition of 2 mL of the organic extraction mixture in the second step, we observed an increase in the volume of the organic phase to about 3.5 mL. On a first glance, this increased volume was thought to be due to an increased water content in the organic phase in the second extraction step. However, experiments with indigo carmine revealed that the water content was actually lower in the second organic phase. The overall water content in the total extraction mixture must be lower in the second step, as a fraction of water was removed in the first step. To improve understanding of the fundamental physicochemical aspects of the SWIEET extraction, further experiments on the composition of the phases will be conducted in the future.

In the first step, average recoveries reached 37% using SWIEET. Notably, in the second step, another 32% were recovered (experiment 9), but the increase in the second step was especially high for polar analytes. For metformin, for example, 21% were recovered in the first step and additional 38% in the second step. It is important to state that for unpolar analytes like DEET and acridine (2.5 < logD_pH7_ < 3.5), recoveries were already high in the first step with 50% each, while in the second step, only an additional 20% was extracted. For the three most polar analytes (− 1.5 > logD_pH7_ >  − 5.7) metformin, EMI, and acesulfame, recoveries were clearly higher in the second step (36–44% extracted) than in the first step (20–34%).

Interestingly, the application of an electric field did not yield highest recoveries, as shown in Fig. 2c and d, where we compared a double-extraction with and without 200 µA applied (experiment 4 vs. 15). We observed high recoveries for positively charged analytes and low recoveries for negatively charged analytes in the first step as would be expected due to the polarity applied. Nevertheless, recoveries achieved without electroextraction exceeded those by 17% in the first step. In the second step, the polarity was reversed, so negatively charged analytes became extracted primarily. On average, however, recoveries were similarly low in both steps (20% and 18%). Combining a regular extraction with electroextraction in the second step (experiment 7) increased overall recoveries to 48% as would be assumed from the previous observations (see the “Electroextraction” section), but still, total recoveries of 55% from diffusional extraction using the same organic extraction mixture were not reached. This might be due to the complex matrix of wastewater effluent used in this set of experiments, as discussed in the “Electroextraction” section.

Overall, the double-extraction using 80–20 acetonitrile-isopropanol as the organic extraction mixture in both steps without the application of an electric field revealed the highest average recoveries (69%), which is similarly high as the QuEChERS extraction (66%). This protocol was chosen to be applied to different types of samples.

### Comparison of SWIEET to QuEChERS for different types of samples

**Fig. 3:**
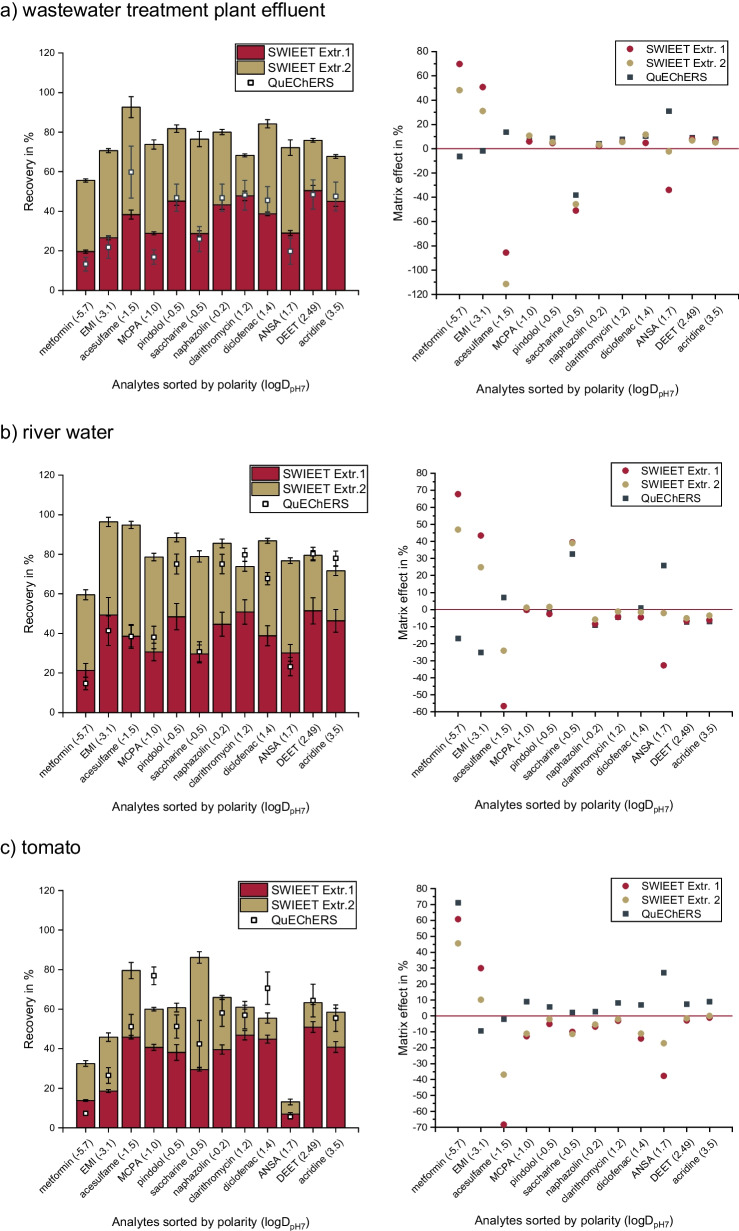

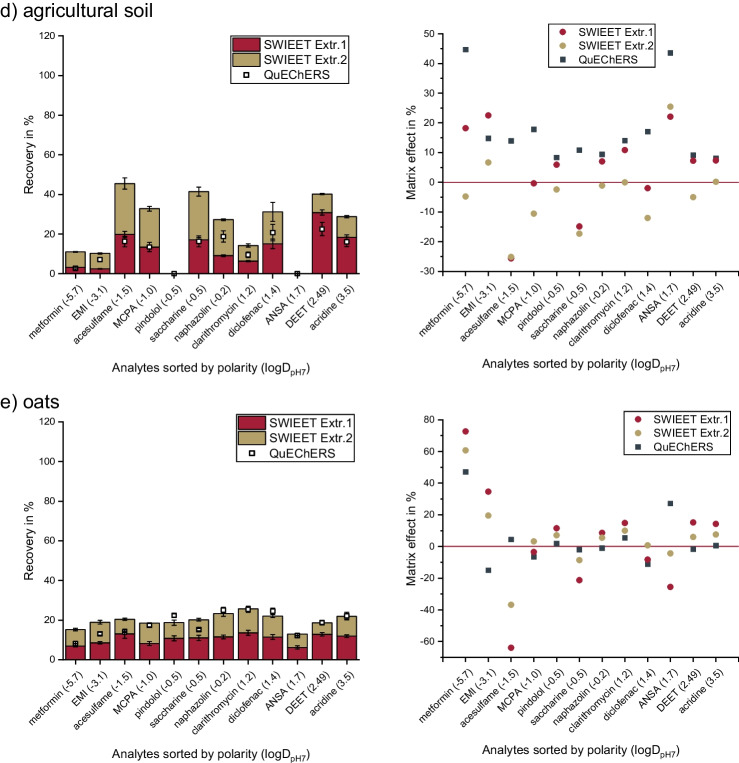
Average recoveries (left) and matrix effects (right) (*n* = 5) for extractions of the analyte mix (3 mg/L) (see the “Model analyte mix” section) when spiked to ***a*** wastewater treatment plant effluent, ***b*** surface water, ***c*** mashed tomato, ***d*** soil, and ***e*** oats using the SWIEET double-extraction compared to QuEChERS. For detailed extraction procedures, see the “Final SWIEET protocol” and “QuEChERS extraction” sections

We compared the SWIEET double-extraction directly to the classical QuEChERS protocol using different liquid and solid samples: wastewater treatment plant effluent, river water, mashed tomato, an agricultural soil, and oats. Both extraction methods were carried out without further clean-up steps. Results are shown in Fig. 3. For all samples, matrix effects were determined (see the “Quantification” section).

Liquid samples/samples with a high water content: For wastewater treatment plant effluent, recoveries increased significantly in the second extraction step for all model analytes. This was prevalent especially for positively charged and highly polar and charged analytes like metformin, EMI, and acesulfame, for which a higher fraction was extracted in the second step than in the first step (see Fig. 3a–c). For all model analytes, the recoveries reached with the SWIEET double-extraction exceeded those reached using QuEChERS. Averaged over all analytes, SWIEET yielded 75%, QuEChERS only 37%. The same double-extraction was applied to surface water samples and tomato. On average, SWIEET yielded 81% and 57% and QuEChERS 54% and 47% for surface water and tomato, respectively. It is also notable that standard deviations were always lower using the SWIEET method, which indicates a higher repeatability compared to QuEChERS (e.g., for tomato, average standard deviation SWIEET 3.7%, QuEChERS 6%). We assume that this is due to the lack of the additional solid phase in the SWIEET method, which reduces possible sorption phenomena.

In river water and wastewater treatment plant effluent, matrix effects in SWIEET were similar to QuEChERS for most unpolar and medium polar analytes. Matrix effects were significantly higher for EMI, acesulfame, and ANSA in SWIEET compared to QuEChERS and compared to less polar analytes. For example, for metformin and EMI, matrix effects were 70% and 51% in wastewater treatment plant effluent for the first SWIEET extraction, compared to − 6% and − 2% for QuEChERS, but a direct comparison is hindered by the 2–4 times lower absolute concentrations in the QuEChERS extract. For acesulfame and diclofenac, matrix effects were also higher for SWIEET, but with ion suppression for SWIEET vs. ion enhancement for QuEChERS. For the tomato sample, differences between QuEChERS and SWIEET were more pronounced for unpolar and medium polar analytes, compared to wastewater treatment plant effluent and river water. For pindolol in wastewater treatment plant effluent, the matrix effect was 9% for QuEChERS and 5% for SWIEET, in tomato 5% and − 5%, respectively. Since the SWIEET method is superior in extracting polar analytes, more polar matrix compounds are likely to be coextracted causing these elevated matrix effects. Better separation, for example, with HILIC, would minimize these effects.

Dry samples: For the extraction from soil, SWIEET by far exceeded QuEChERS recoveries with 24% compared to 12%. Lowest recoveries were achieved for the extraction from oats with 20% and 18% both for SWIEET and QuEChERS. Higher recoveries than those achieved by us are possible with QuEChERS for similar samples, as shown by de Matos et al. [46] and Michel et al. [47]. Since we achieved low recoveries with both extraction methods, the problem is assumed to be due to the preparation of the solid samples. Since matrix effects were similar compared to tomato or aqueous sample extraction, a problem in LC–MS analysis is less likely. For sample preparation of the solid samples, water was added to the dry sample to create a slurry which was spiked with the analyte mix, mixed for 1 h using an overhead shaker and then dried in the oven at 60 °C. This was done to assure that the analytes are (partly) sorbed on the solid phase and not only dissolved in the added water, which would facilitate extraction. Possibly, the sorption of the analytes was not fully overcome by the extraction methods used. Alternatively, thermal degradation or evaporation may have occurred.

**Fig. 4:**
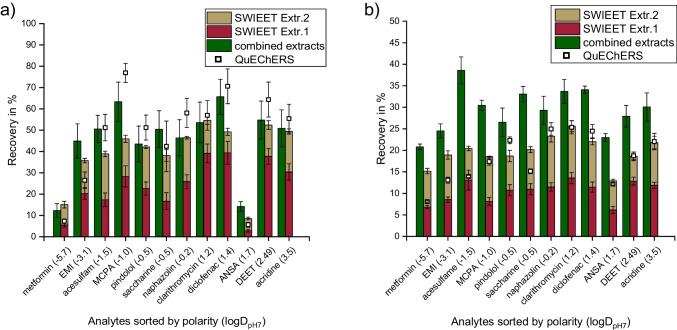
Recoveries of the model analytes (see the “Model analyte mix” section) after QuEChERS or SWIEET double-extraction from spiked ***a*** mashed tomato and ***b*** oats (see the “Spiking of dry samples” section). Red and gold bars indicate extracts that were analyzed individually and green bars that the analytes were quantified in the combined extracts. For detailed extraction procedures, see the “QuEChERS extraction” and “Final SWIEET protocol” sections

Combination of the extracts from double-extraction: Since in SWIEET recoveries were similar in the first and second step for most analytes and sample types, extracts from both steps were combined and analyzed, as no significant dilution occurs upon combining the extracts. Figure 4 shows the direct comparison of the recoveries from the combined extracts to the individual extracts using oats as sample. Higher average recoveries were achieved with the analysis of the combined extract (29%) than with the sum of the individual extracts (10% + 10% = 20%). This is likely due to a reduction of matrix effects, since only one sample has to be analyzed instead of two. This finding was confirmed with the extraction of a mashed tomato, where we achieved 46% with the combined extracts and 40% with the individual extracts. The combination of the extracts not only facilitates the extraction protocol and reduces analysis time, but it also increases the total analyte recovery. As seen in Fig. 4, extracts exceed those achieved with QuEChERS for oats and were similar for the extraction of a tomato.

Overall, recoveries and matrix effects determined for SWIEET compared well or are better than those reached with QuEChERS for the matrices tested. Especially for polar analytes like metformin, EMI, and acesulfame, SWIEET surpasses QuEChERS for all sample types. We saw matrix effects especially in case of polar analytes, presumably due to co-extraction of matrix components. Cleanup strategies, e.g., with dispersive solid-phase extraction will have to be implemented in the future. SWIEET can be applied successfully to aqueous samples or samples with high water contents, but also to solid samples, where it yielded similar or higher recoveries as the QuEChERS extraction. Aqueous samples may require the addition of solid glucose instead of concentrated glucose solutions to reduce the volume of the extraction medium including the organic phase. We are not yet satisfied with the extraction efficiencies of solid samples, where further work is intended. In the future, the application of the method will be broadened to address further types of samples. With the simplified extraction protocol, SWIEET could improve sample preparation for analytical chemists working with environmental, food, or biological samples up to body fluids. SWIEET is of particular interest in biota analysis as the reduced amount of solid phase may allow to use smaller extraction volumes compared to QuEChERS.

## Conclusion

We successfully developed a new extraction method combining sugaring-out with the addition of a protic organic solvent with a double-extraction. By screening a variety of possible additives for the extraction, we found that sugars helped to induce a very robust phase boundary over a wide temperature range and provided relatively high recoveries for our broad mixture of analytes. With glucose, we identified a cheap and readily available additive, which is highly soluble and therefore easy to handle and can simply be added by pipetting. Recoveries were improved from 23 to 34% comparing extractions with and without sugar.

To further improve recoveries, we investigated the influence of the volume and composition of the extraction mixture on the extraction recoveries. Adding isopropanol as a polar protic solvent resulted in the highest recoveries for polar analytes and also increased repeatability, as indicated by the lower standard deviations. It is also a great non-toxic alternative to halogenated solvents used in other studies.

Further improvements were based on a DoE including temperature, isopropanol, and glucose content. Since recoveries did not exceed 50%, we tested electroextraction and double-extraction for further optimization. Electroextraction improved recoveries to over 60% for most analytes. Especially charged analytes like metformin, for which 32% were recovered, profited compared to 6% recovered without electroextraction. However, for real samples with higher salt loads, electroextraction was not successful.

Double-extraction significantly improved the recoveries of the SWIEET method. For QuEChERS, we only saw an improvement for single analytes like EMI and ANSA. Overall, using the SWIEET double-extraction, similar recoveries as with the regular QuEChERS extraction were often reached. For very polar substances, SWIEET clearly outperformed QuEChERS. Double-extraction is fast compared to weighing the salts and centrifuging the samples in QuEChERS. Also, handling is facilitated since all components are in solution when liquid samples are extracted and no solid phase impairs recoveries.

We applied the SWIEET method to aqueous and dry model samples. SWIEET proved to be similarly applicable as QuEChERS and yielded comparable or higher recoveries for the model analytes, while maintaining high repeatability. However, for wastewater treatment plant effluent, 75% recovery on average was achieved with SWIEET, compared to 37% with QuEChERS. Future work will address modifications of the original QuEChERS method such as QuEChERSER but also QuPPe.

Our results show that SWIEET is an interesting alternative to QuEChERS. All components used are cheap and readily available, and toxicity is low. Handling is facilitated, since tedious weighing of salts is replaced with pipetting. We showed that the SWIEET method, a salt-free extraction method, works for a broad range of sample matrices for the extraction of analytes widely differing in polarity, size, charge, and functional groups. This makes the method an interesting alternative that should be tested for further applications.

## Supplementary Information

Below is the link to the electronic supplementary material.

Supplementary file1 (DOCX 657 KB)
