# Supplementary material for: SWIEET—a salt-free alternative to QuEChERS
Source: Anal Bioanal Chem. 2024 Sep 18;416(28):6387–403. doi: 10.1007/s00216-024-05525-0 (PMC11541295; doi:10.1007/s00216-024-05525-0)
Supplement: Supplementary file 1 — Supplementary file1 (DOCX 657 KB) [file 216_2024_5525_MOESM1_ESM.docx]

## **Supporting Information to**

## **“SWIEET — A salt-free alternative to QuEChERS”**

Nadja Kalinke, Pascal Stopper, Luca Völkl, Florian Diehl, Carolin Huhn*

Institute of Physical and Theoretical Chemistry, Department of Chemistry, Eberhard Karls Universität Tübingen, Tübingen, Germany

*corresponding author: carolin.huhn@uni-tuebingen.de

### S1 Choice of the organic solvent

The halogenated solvents dichloromethane and chloroform were used by Liu et al [1] to induce phase separation of water and acetonitrile, the system yielded high recoveries of 100% for glycosides and aglycones. In our study, we compared the extractions using chloroform, dichloromethane, EtOAc and isopropanol. A notably larger phase ratio of the organic:aqueous phase was observed after the addition of isopropanol. Among the organic solvent additives, the protic isopropanol is more strongly excluded from the aqueous phase. A larger organic phase volume indicated that the organic phase is more polar (see Section “Choice of Organic Solvent” in the main text), which we would expect to result in higher recoveries especially for polar analytes, further aided by the large volume of the organic phase. The disadvantage of this large volume of the organic phase is the higher dilution.

**Fig. S1** Recoveries of the model analytes after extraction using 90-10 vol.% acetonitrile-solvent in the organic extraction mixture. Solvents chosen were chloroform, dichloromethane, EtOAc and isopropanol. The aqueous extraction mixture consisted of 3 mg/L analyte mix (see “Model analyte mix”) and 2 M glucose in doubly-distilled water. For the detailed extraction procedure, see “Optimization of SWIEET extraction procedure” in the main text

Looking at analyte recoveries in Fig. S1, especially for unpolar and medium polar analytes (logD_pH7_ > 0) the addition of chloroform resulted in the highest recoveries, followed by DCM. Recoveries using EtOAc and isopropanol were similar but lower for this class of analytes. For polar analytes, however, with logD_pH7_ ≤ −0.5, the recoveries improved with isopropanol compared to chloroform, especially for the most polar analytes EMI and metformin, which were hardly extracted (only 12 and 1% for chloroform) when adding the aprotic, non-polar solvents. For MCPA, which is also polar, recoveries followed the order EtOAc (77%) >> isopropanol (34%) ~ dichloromethane (34%) > chloroform (23%). For acesulfame (logD_pH7_ = −1.7), the use of chloroform resulted in about 30% higher recoveries compared to the other solvents, but the reasons for this enhancement are not clear.

Isopropanol was the only solvent tested, that was not only polar, but also protic. We assume that it not only increased polarity in the organic phase, but also enabled better hydrogen bonding in the organic phase. The increase in recoveries for strongly polar and especially the charged analytes can be explained by enhanced solubilization in the organic phase aided by H-bond formation. Another factor, that has an influence on the extraction is the permittivity. Isopropanol has the highest permittivity of all the organic solvents tested as additives, expected to increase the solubility for polar and ionic solutes [2], which is in line with our observation.

Since the focus of this work was to improve the extraction of polar and ionizable analytes, while maintaining high recoveries of unpolar analytes, we chose isopropanol for further optimization.

### S2 Screening of additives to improve phase separation

Some additives tested in Section “Screening of additives to induce phase separation” in the main text produced signals in LC-MS, that partially overlapped with analyte signals. This can lead to suppression of the analyte signal. To minimize the effect that this has on the evaluation of the additive during our screening approaches, we used *median* recoveries for the screening. Using sugars, only the very polar analytes ANSA and acesulfame partially coeluted, which is why *average* recoveries were used in the following. Fig. S2 shows the extracted ion chromatograms of the model analytes after extraction with glucose. The glucose added in SWIEET extractions stays mainly in the aqueous phase. Only a small amount is detected in the organic phases in ESI− mode, but it elutes in the dead volume and therefore does not affect ionization of the analytes significantly. In ESI+, only the most polar analytes may become affected, but it has to be noted, that RPLC is anyhow not optimal for the separation of these very polar analytes. Separation can be improved by using HILIC or SFC.

Since sugars proved to be successful as additives, recoveries were compared in detail in Fig. S3.

| a) ESI+ |
| --- |
| 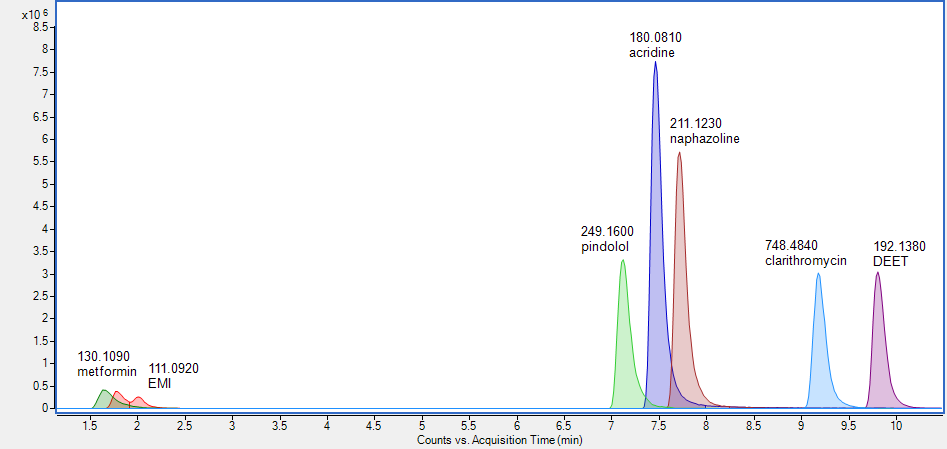 |
| b) ESI- |
| 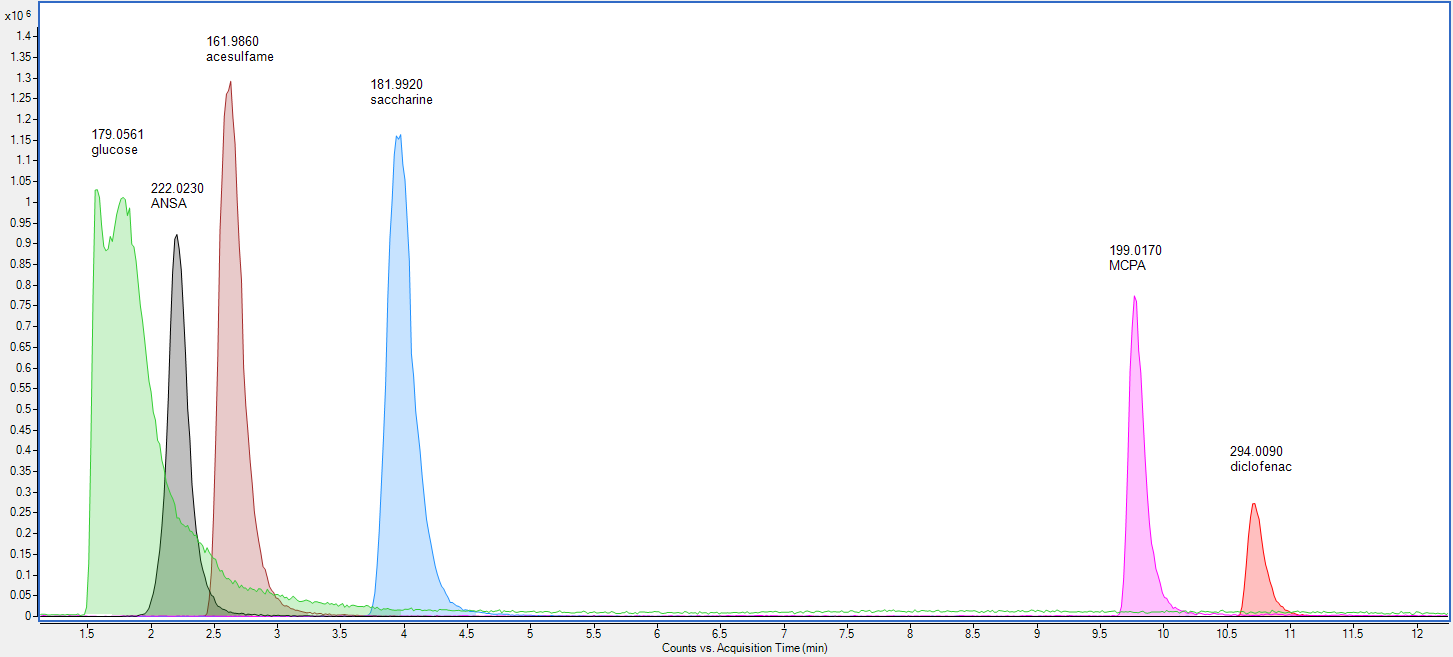 |

**Fig. S2** Extracted ion chromatograms of glucose and analytes detected in a) ESI+ and b) ESI-. Measurement of the first organic phase from a SWIEET extraction of surface water, spiked post extraction with 0.42 mg/L model analyte mix. For the detailed extraction protocol, see “Final SWIEET protocol” in the main text. For LC-MS parameters, see “LC-MS method” in the main text. For details on the analyte mix, see “Model analyte mix” in the main text


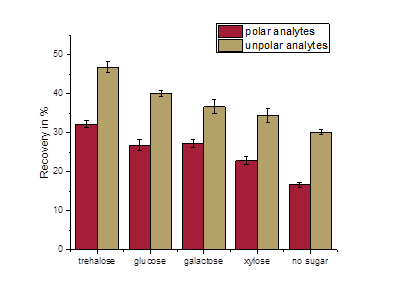


**Fig. S3** Average recoveries of all model analytes (see “Model analyte mix”) from three replicates depending on the type of sugar added to the aqueous phase at a concentration of 1 M. The organic solvent mixture consisted of 65% MeCN and 35% EtOAc. For details on the extraction procedure, see “Optimization of the SWIEET extraction”

Higher sugar concentrations increased partition and distribution coefficients of three organic molecules in a study by Wang et al. [3]. Glucose addition yielded highest recoveries for their analytes in the logD_pH7_ range of -1.23 to -0.89. For our broad analyte spectrum, it ranked similar to galactose and second to trehalose. However, our recoveries were <50% necessitating further optimization. We preferred glucose, since it is significantly cheaper than trehalose while recoveries were acceptable. Its solubility is higher than that of galactose, which enables to use a broader range of additive concentrations to adapt phase separation and polarity for specific analytical tasks. Interestingly, upon addition of glucose, EtOAc was no longer necessary to guarantee a stable phase separation and this solvent was omitted in further optimization steps.

### S3 Optimization of extraction parameters

As discussed in the main text, the extraction parameters isopropanol and glucose concentration, as well as temperature were optimized using a design of experiment. Results were displayed as hypersurface plots in Fig. S4.

| a) | b) |
| --- | --- |
| 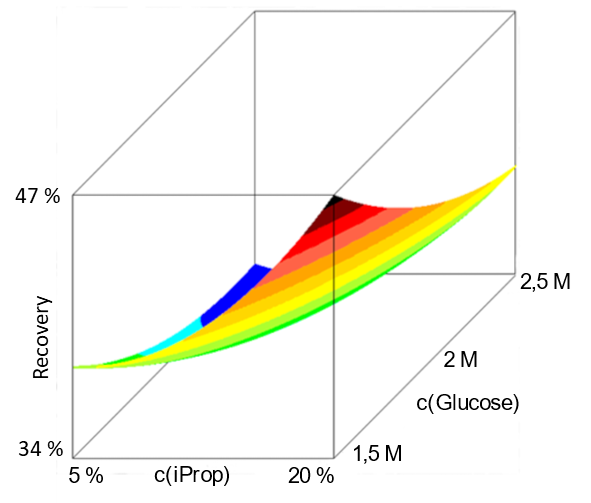 | 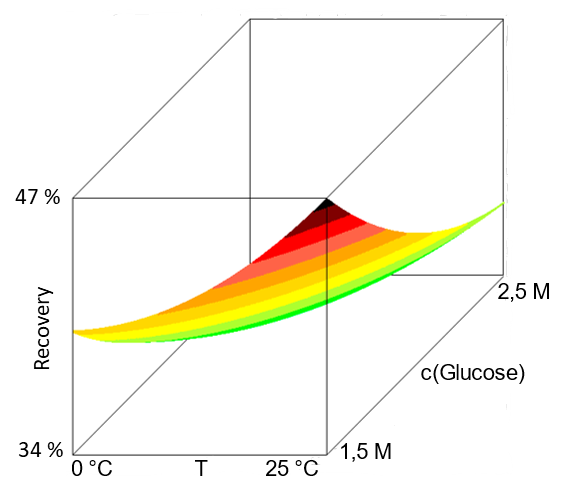 |
| c) | |
| 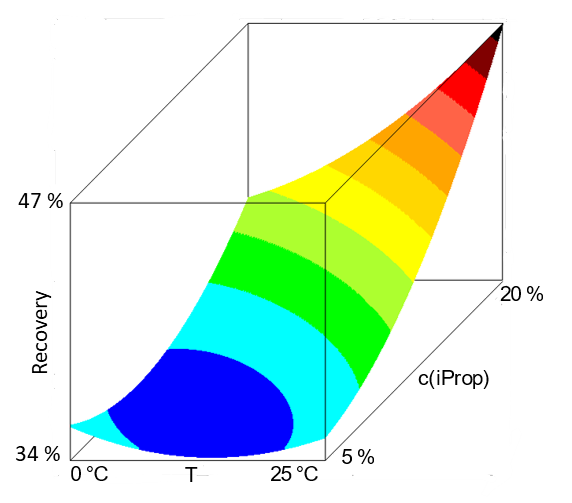 | |

**Fig. S4** Surface plots of the average recoveries of all analytes determined from the DoE (see Materials and Methods), in dependence of a) isopropanol and glucose concentrations, b) glucose concentrations and temperature and c) temperature and isopropanol concentrations. Sample: aqueous extraction mixture

### S4 Electroextraction

Electroextractions were conducted to increase recoveries for ionic and ionizable analytes. To conduct these experiments, a syringe was modified as shown in Fig. S5. Two platinum electrodes were introduced into the syringe to enable the application of an electric field to the extraction mixture. Due to the clear plastic of the syringe, the phase boundary could be monitored during extraction, as well as possible bubble formation caused by electrolysis. Fig. S6a shows the improved recoveries with the application of an electric field, compared to the extraction without it. Fig. S6b compared electroextractions conducted in doubly distilled water and WWTP effluent, demonstrating the strong impact of the (ionic) matrix load on recoveries.


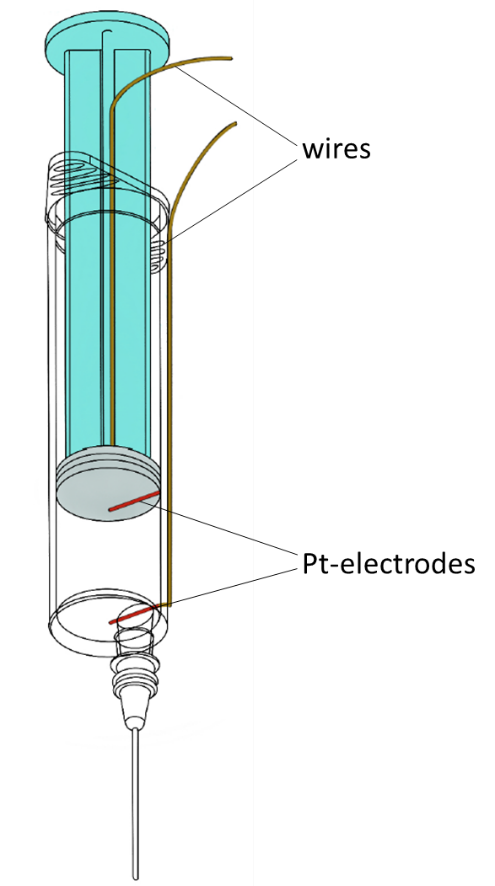


**Fig. S5** Setup of the syringe modified for electroextraction. A regular plastic syringe was equipped with two platinum electrodes at the stamp and the outlet, which were then connected to a high voltage power supply

|   a) |   b) |
| --- | --- |

**Fig. S6** Recoveries of all 13 model analytes (see Section “Model analyte mix”) after 10 min extraction applying 200 µA or 0 µA. Aqueous phase: a) doubly distilled water b) doubly distilled water or WWTP effluent; organic phase: acetonitrile-isopropanol 80:20; 2 M glucose added. For the electroextraction protocol, see Section “Electroextraction”. Charge number at pH 7 for analytes given in brackets

### S5 Double-extraction with/without electroextraction

As described in the main text, 15 double-extractions were conducted with varying volumes, phase compositions and electric fields. The conditions are provided in Table 3 in the main text. The results of the experiments are shown in Fig. S7. Results are discussed in the main text.

| 1 | 2 |
| --- | --- |
|  |  |
| 3 | 4 |
|  |  |
| 5 | 6 |
|  |  |
| 7 | 8 |
|  |  |
| 9 | 10 |
|  |  |
| 11 | 12 |
|  |  |
| 13 | 14 |
|  |  |
| 15 |  |
|  |  |

**Fig. S7** Recoveries for the double-extraction experiments of the analyte mix (see Section “Model analyte mix”) in WWTP-effluent. Extraction parameters 1-15 are summarized in Table 3 in the main text. The currents set for electroextraction as well as the organic extraction mixture composition acetonitrile-isopropanol are given in the panels. For the detailed extraction protocol see Section “Optimization of the SWIEET extraction protocol” and “Electroextraction” in the main text

1. Liu G, Yang H, Zhang M, Li S, Chen B, Yao S. Novel isolation of phytochemical compositions by phase transition extraction with acetonitrile. Journal of Separation Science. 2011;34(3):347-53. <https://doi.org/10.1002/jssc.201000658>.

2. Liao X, Raghavan VGS, Meda V, Yaylayan VA. Dielectric properties of supersaturated α-D-glucose aqueous solutions at 2450 MHz. Journal of Microwave Power and Electromagnetic Energy. 2001;36(3):131-8. <https://doi.org/10.1080/08327823.2001.11688455>.

3. Wang B, Ezejias T, Feng H, Blaschek H. Sugaring-out: A novel phase separation and extraction system. Chemical Engineering Science. 2008;63(9):2595-600. <https://doi.org/10.1016/j.ces.2008.02.004>.
